# A multidisciplinary opioid-reduction pathway for robotic prostatectomy: outcomes at year one

**DOI:** 10.1186/s13741-023-00331-1

**Published:** 2023-08-01

**Authors:** Michael W. Manning, John Whittle, Matthew Fuller, Sara H. Cooper, Erin L. Manning, Joe Chapman, Judd W. Moul, Timothy E. Miller

**Affiliations:** 1 Department of Anaesthesiology, Duke University, Durham, NC 27710 USA; 2 Centre for Perioperative Medicine, Division of Surgery & Interventional Science, UCL, London, UK; 3 Department of Biostatistics, Duke University, Durham, NC USA; 4 Department of Pharmacy, Duke University, Durham, NC USA; 5 Department of Surgery, Duke University, Durham, NC USA

**Keywords:** ERAS, Opioid, Opioid-free anaesthesia

## Abstract

**Background:**

Opioid use has come under increasing scrutiny, driven in part by the opioid crisis and growing concerns that up to 6% of opioid-naïve patients may become chronic opioid users. This has resulted in a revaluation of perioperative practice. For this reason, we implemented a multidisciplinary pathway to reduce perioperative opioid usage through education and standardization of practice.

**Methods:**

A single-centre retrospective evaluation was performed after 1 year, comparing the outcomes to those of the 2 years prior to pathway implementation. Comparisons were made between pre- vs. post pathway change by 2:1 propensity matching between cohorts. Univariate linear regression models were created using demographic variables with those that were *p* < 0.15 included in the final model and using post-operative opioid use (in oral morphine equivalents, OME) as the primary outcome.

**Results:**

We found that intraoperative opioid use was significantly decreased 38.2 mg (28.3) vs. 18.0 mg (40.4) oral morphine equivalents (OME), *p* < .001, as was post-operative opioid use for the duration of the hospitalization, 46.3 mg (49.5) vs. 35.49 mg (43.7) OME, *p* = 0.002. In subgroup analysis of those that received some intraoperative opioids (*n* = 152) and those that received no opioids (*n* = 34), we found that both groups required fewer opioids in the post-operative period 47.0 mg (47.7) vs. 32.4 mg (40.6) OME, *p* = 0.001, + intraoperative opioids, 62.4 mg (62.9) vs. 35.8 mg (27.7) OME, *p* = 0.13, - intraoperative opioids. Time to discharge from the PACU was reduced in both groups 215 min (199) vs. 167 min (122), *p* < 0.003, + intraoperative opioids and 253 min (270) vs. 167 min (105), *p* = 0.028, - intraoperative opioids. The duration of time until meeting discharge criteria from PACU was 221 min (205) vs. 170 min (120), *p* = 0.001. Hospital length of stay (LOS) was significantly reduced 1.4 days (1.3) vs. 1.2 days (0.8), *p* = 0.005. Both sub-groups demonstrated reduced hospital LOS 1.5 days (1.4) vs. 1.2 days (0.8), *p* = 0.0047, + intraoperative opioids and 1.7 days (1.6) vs. 1.3 days (0.9), *p* = 0.0583, - intraoperative opioids. Average pain scores during PACU admission and post-PACU until discharge were not statistically different between cohorts.

**Conclusions:**

These findings underscore the effectiveness of a multidisciplinary approach to reduce opioids. Furthermore, it demonstrates improved patient outcomes as measured by both shorter PACU and almost 50% reduction in perioperative opioid use whilst maintaining similar analgesia as indicated by patient-reported pain scores.

**Supplementary Information:**

The online version contains supplementary material available at 10.1186/s13741-023-00331-1.

## Introduction

Perioperative opioid use has come under increasing scrutiny, driven largely by worsening trends in opioid-related adverse events (ORAEs) (Brummett et al. 2017; Rudd et al. 2016; Kharasch et al. 2020), including observations that higher levels of opioid use during surgery result in increased opioid use in the post-operative period to maintain equivalent levels of analgesia (Chia et al. 1999; Hayhurst and Durieux 2016; Collard et al. 2007). Continued use of opioids at these higher amounts or as a single agent for analgesia during surgery is therefore inconsistent with overall trends to limit the impact of opioids and may in fact result in increased overall opioid use, worse post-operative pain, and decreased patient satisfaction.

Moreover, there are growing concerns around the observation that up to 6% of opioid-naïve patients across all ages will become chronic opioid users, as defined by continued use ≥ 90 days or repeated refills of prescription following surgery (Brummett et al. 2017; Reuben et al. 2015). Furthermore, mounting fears of diversion and addiction associated with overprescribing (Reuben et al. 2015) have induced many practitioners to re-evaluate their individual administration and prescribing practices (Koepke et al. 2018). Yet, despite implementation of stricter prescribing laws limiting discharge opioids, there remains a disconnect between opioid use in the perioperative period and those given at the time of discharge. Recent consensus statements from the Perioperative Quality Initiative (POQI) on opioid minimization in opioid-naïve patients help to set a standard and outlines several non-opioid analgesics that can be utilized to help minimize opioids during the perioperative period (Wu et al. 2019).

We conducted an internal audit of our own institutional intraoperative opioid utilization and noted that there was great provider variability in both practice and dosing. In those patients undergoing laparoscopic cholecystectomy procedures, we observed that the pre-, intra-, and post-operative administration of opioids was highly variable with administration differing among practitioners in both doses (when considered by weight-based dosing or considerations for age) and timing. In 50 cases reviewed, we observed patients receiving on average 250 mcg of fentanyl and almost 2 mg of hydromorphone during an average case length of 103 min. Moreover, the use of non-opioid analgesics was inconsistent and largely considered an ‘afterthought’ (author’s unpublished data). This variability in practice may further contribute to the opioid crisis (Brummett et al. 2017) and makes opioid reduction and opioid stewardship (McEvoy et al. 2017) an attractive option for targeted intervention.

Methods and techniques designed to provide opioid-reduced/opioid-free anaesthesia (OR/OFA) have been reported in the literature (Bakan et al. 2015; Beloeil 2019; Samuels et al. 2017), across different types of surgery with some success (Chanowski et al. 2019). Some authors have reported that opioid use in the post-operative period is unchanged following ORA/OFA, although the absence of a change in opioid use or prescribing practices in the post-operative period may be multifactorial, such as unaltered prescribing practices in the post-operative period including at the time of discharge (Brandal et al. 2017; Soffin et al. 2019).

Guided by observations made during our initial review of current practices, we began implementation of a comprehensive care redesign modelled on published consensus guidelines (McEvoy et al. 2017). The purpose of the collaborative quality improvement project described here was to specifically apply a multidisciplinary approach with an intent to reduce perioperative opioid use whilst maintaining excellent perioperative analgesia and patient care within an established enhanced recovery framework. We used both education and practice guidance protocols, with the goal of improving outcomes. Specifically, we sought to the following: (1) reduce opioid use, (2) decrease opioid-induced side effects and improve post-operative recovery as measured by (3) decreased post-anaesthesia care unit (recovery/PACU) length of stay (LOS), and (4) hospital LOS, starting with those patients undergoing robotic prostatectomies.

Here, we report the first-year outcomes following the implementation of this multidisciplinary, opioid-reduced/opioid-free (OR/OF) pathway.

## Methods

### Pathway design and implementation

**Fig. 1:**
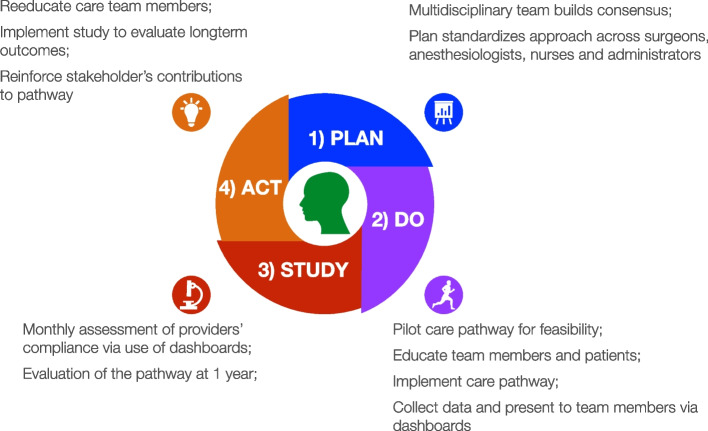
Developmental process for ORA/OFA pathway

Based on the desire to improve patient care and provide a more standardized approach across practitioners, we established an anaesthesiology-led, multidisciplinary group comprised of urological surgeons; nurse practitioners; nurses from the surgery clinics, PACU, and hospital wards; and pharmacists, in order to design our analgesic care pathway for robotic prostatectomies (see Fig. 1). We employed a multidisciplinary group institutional expertise across all domains of patient care during development of the pathway (McEvoy et al. 2017). The pathway contains several key elements; among those are as follows: the establishment of guidelines and expectations for prescribing and treating acute surgical pain in these patients for both practitioners and patients and the use of evidence-based, non-opioid medications first, consistently given at appropriate doses and at regular intervals beginning the day prior to surgery and continuing throughout the surgical continuum until discharge (Table 1).

**Table 1:** Key elements of the multidisciplinary analgesic care pathway

Provide adequate patient education about their procedure and expected outcomes
Establish expectations for post-operative pain and achievable pain management goals for the patient based on patient-provided feedback
Establish mutual expectations for post-operative pain and agree on achievable pain management goals for patients and with those team members caring for them
Understand what medications are available to give preoperatively for pain control
Establish the priority administration of non-opioid analgesics during the intraoperative phase over opioid analgesics
Provide thoughtful and rapid assessment of patient comfort in the PACU whilst providing patients and their family with a review of previous education and expectations
Schedule non-opioid analgesics within this framework, with opioids given as rescue medications available PRN

This pathway went into practice on August 1, 2017, following 1 month of education in the form of formal presentations, lectures, and written communication. We confirmed wide-spread understanding and adoption among the stakeholders through continual education and support during implementation and establishment. The pathway then became the standard of practice for patients undergoing robotic prostatectomies at our institution.

### Review of pathway results

After obtaining internal review board approval for a quality improvement review, we surveyed the medical records of all patients that underwent robotic prostatectomy at our institution between September 1, 2017, and September 1, 2018. Data from the medical records for patients undergoing the same procedure between July 1, 2015, and July 31, 2017, were reviewed and used as a comparison for post-implementation care. Demographic data was collected on all patients in addition to factors that commonly associate with the perception of pain, including histories of chronic pain, anxiety, depression, and prior drug use (Table 2).

**Table 2:** Demographics and characteristics of pre- to post-protocol of all patients

Matched characteristics	Pre (*n* = 469)	Post (*n* = 191)	Total (*n* = 660)	*p*-value
**Age**	61.9 ± 7	61.5 ± 7	61.8 ± 7	0.5070*
**Race**				0.2244†
Black	101	53	154	
Other	45	18	63	
White	323	120	443	
**BMI**	28.7 ± 4.4	29.4 ± 4.8	28.9 ± 4.6	.0244*
**BSA**	2.13 ± 0.2	2.17 ± 0.2	2.14 ± 0.2	.0196*
**ASA score**				0.6552†
1	10	3	13	
2	224	86	310	
3	233	102	335	
4	2	0	2	
**Smoker**	42	33	75	.0042‡
**Chronic pain (current)**	36	17	53	0.6362‡
**Chronic pain**	47	32	79	.0239‡
**Anxiety**	14	10	24	0.1723‡
**Major depression**	13	9	21	0.2331‡
**Mood disorder**	0	0	0	N/A
**Drug use disorder**	1	4	5	.0265‡
**Opioid tolerant**	1	2	3	0.2024‡
**Sleep apnoea**	43	23	66	0.3162‡

^*^Wilcoxon, †chi-square, ‡Fisher exact (values reported as mean ± STD)

We hypothesized that post-operative outcomes, specifically post-operative opioid use and incidence of PON/V, would be improved through adaptation of this multidisciplinary approach to opioid reduction. By addressing this hypothesis, we hope to inform potential interventions that will result in both reduced opioid use and in improved patient outcomes.

### Pre-implementation

Prior to implementation of the care redesign, we found perioperative care was conducted on a case-by-case bases. Therefore, each case was highly variable resulting from great individuality among practitioners, specifically regarding premedication administration, intraoperative opioid use, and management of post-operative pain (author’s unpublished data). Patients were not consistently nor uniformly instructed about expectations for their recovery and the anticipated pain levels after the procedure. Multimodal, non-opioid analgesic use was infrequent and sporadic among the care team, during all phases of care.

### Protocol for consistency of pain management

We previously published our protocols for opioid-reduction/opioid-free anaesthesia (see Supplement Table 1) (Koepke et al. 2018), and these protocols were adapted specifically for patients undergoing robotic prostatectomy, where focus was directed to providing a consistent practice approach.

**Fig. 2:**
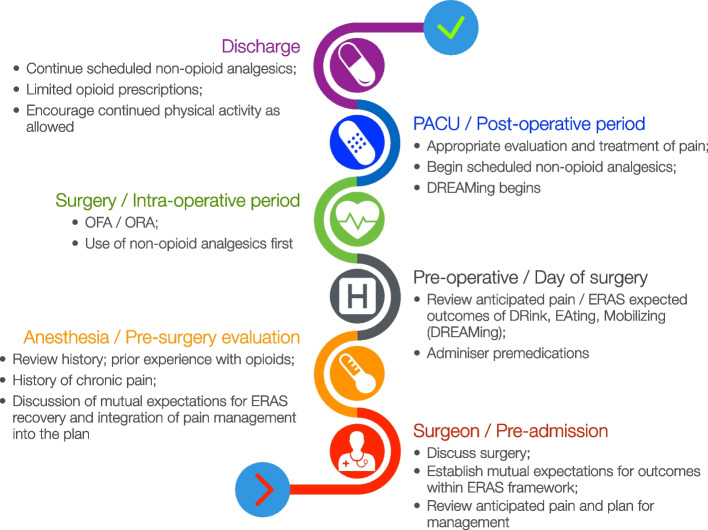
Timing and locations of planned interventions for opioid reduction in robotic prostatectomies

Patient education and the alignment of patients’ expectations for post-operative analgesia were central to the care pathway (see Supplement Table 2). During the care redesign, representatives from every domain of the patient’s care continuum participated. Expectations were set for patient outcomes, and all stakeholders discussed what they would be able to contribute to and support the outcome goals. Once consensus was met, the care redesign was distributed, and education of the care teams was performed (Fig. 2).

The collaborative pathway that was developed by consensus (included in full on the Online supplement) was distributed to the various team members along the surgical continuum for education and review. Points were clarified to the team members through a series of lectures and presentations in the month before implementation. Additional small group meetings were held during the first month of implementation as check-in and to address questions and concerns as related to workflow and acceptance by the various care team members. Dashboards were developed to extract metric data from the medical records on a weekly basis for evaluation of compliance. A goal of 80% compliance was targeted for the pathway; however, year-long compliance was better, with the monthly average consistently maintained > 90%.

### Statistical analysis

We conducted propensity matching 2:1 of patients from 2 years prior to the implementation of this care pathway to patients after 1 year of the pathway. Univariate linear regression models were created with post-operative opioid use (in oral morphine equivalents, OME) as the outcome and each of the demographic variables above as well as intraoperative opioids and time (pre vs. post pathway change). Any variable *p* < 0.15 or less was included in the initial multivariable model. Backward selection was used with AIC to identify the final multivariable model.

All variables except BMI and BSA had a *p*-value < 0.15 in the univariate analysis and were included in the initial multivariable model. The final multivariable model includes time (pre vs. post), intraoperative opioid use, age, race, ASA score, smoking status, chronic pain, history of chronic pain, and history of anxiety. The SQUIRE checklist was used in the preparation of this manuscript (Ogrinc et al. 2016).

## Results/outcomes

We report the outcomes of a retrospective study in 191 patients undergoing robotic prostatectomy, following the first year after implementation of a multidisciplinary, quality-improvement project. The specific goal of this process was reducing variability in and overall use of opioids across the surgical continuum for these patients through the development of a standardized approach to opioid use.

In the matched data of all patients that underwent the new pathway to those in the 2 years prior to implementation, we found that average intraoperative opioid use was significantly decreased (38.2 mg vs. 18.0 mg OME, *p* < 0.001; Table 3). Pain scores between cohorts were not statistically different when examining the period immediately postoperatively during their PACU admission, nor was there any difference during the remainder of the hospitalization (Table 3).

**Table 3:** Composite outcomes of all patients (receiving opioids + opioid-free)

Outcomes	Pre (*n* = 469)	Post (*n* = 191)	Total (*n* = 660)	*p*-value
**Intraoperative opioids** *(OME)*	38.2 (28.3)	18.0 (40.4)	32.4 (33.5)	.0001*
**Post-operative opioid use** *(OME)*	46.3 (49.5) 31.8 [10.0, 64.5]	35.54 (43.7) 20.0 [5.0, 50.0]	43.2 (48.1) 29.7 [7.5, 61.27]	.0021*
**Hospital LOS (days)**	1.4 (1.3)	1.2 (0.8)	1.4 (1.2)	.0050*
**Post-op antiemetics**				
Any (y/n)	169	54	223	.0559†
Av**era**g**e** no. of doses	0.71 (1.3)	0.43 (0.9)	0.63 (1.2)	.0163†
**PACU LOS (minutes)**	221 (205)	170 (120)	206 (186)	.0001*
**Average pain score**				
**In PACU**	3.6 [2, 5]	3.9 [2.3, 5.4]		0.287*
**On ward**	3.0 [1.8, 4.2]	3.0 [1.6, 4.1]		0.567*

^*^Wilcoxon, †chi-square (values reported as mean (STD) and median [IQR]

Post-operative opioid administration, which remained available to all patients PRN, was significantly reduced over the entire duration of the hospitalization (46.3 mg ± 49.5 vs. 35.5 mg ± 43.7 OME, *p* = 0.002). The opioids used within the post-operative period included hydromorphone, fentanyl, oxycodone, morphine, and methadone.

Post-operative nausea and/or vomiting was assessed using the outcome of any post-operative administration of antiemetics. Any use of these antiemetic medications, including use of ondansetron, metoclopramide, diphenhydramine, famotidine, haloperidol, and promethazine, constituted a ‘yes’ vs. ‘no’ antiemetic use where approximately 51.5% needed at least 1 dose (pre vs. 26.5% post; *p* = 0.0163; Table 5).

Patients who received any doses of opioids intraoperatively saw a nonsignificant reduction in antiemetic use (105 vs. 43 patients, *p* = 0.1259) vs. those who did not receive intraoperative opioids (35 vs. 9 patients, *p* = 0.0163; Table 4)). In this study, we were able to capture evidence of post-operative nausea/vomiting, but additional side effects such as pruritus or gastrointestinal complications were not directly captured into the EMR for retrieval in a retrospective manner. We can only speculate that if these events did occur, they did so at a level that did not contribute to worse outcomes as evidenced by increased PACU or hospital lengths of stay.

**Table 4:** Patients receiving reduced intraoperative opioids (pre to post)

**Matched data**	**Pre (*n* = 296)**	**Post (*n* = 152)**	**Total (*n* = 448)**	*p*-value
**Age**	61.6 (7.1)	61.6 (7.0)	61.6 (7.0)	0.8755*
**Race**				0.5068†
Black	64	39	103	
Other	27	16	43	
White	205	97	302	
**BMI**	29.5 (4.6)	29.5 (4.7)	29.5 (4.7)	0.6424*
**BSA**	2.2 (0.21)	2.2 (0.21)	2.2 (0.21)	0.4308*
**ASA score**				0.9144†
1	6	3	9	
2	131	68	199	
3	158	81	239	
4	1	0	1	
**Smoker**	35	24	59	0.2416‡
**Chronic pain (current)**	17	10	27	0.8342‡
**Chronic pain**	34	20	54	0.6466‡
**Anxiety**	11	7	18	0.6217‡
**Depression**	11	5	16	1‡
**Mood disorder**	0	0	0	N/A
**Drug use disorder**	1	1	2	1‡
**Opioid tolerant**	1	1	2	1‡
**Sleep apnoea**	29	15	44	_1‡_
**Intraoperative opioid use** *(OME)*	39.7 (33.4)	22.1 (44.3)	33.73 (38.31)	.0001*
**Hospital LOS** *(days)*	1.5 (1.4)	1.2 (0.8)	1.4 (1.2)	.0047*
**Post-o**p** antiemetics**				
**Any (Y/N)**	105	43	148	0.1259†
**Average no. of doses**	0.72 (1.4)	0.44 (0.9)	0.63 (1.3)	.0640*
**Post-o**p** opioids** *(OME)*	47.0 (47.7) 34.5 [11.7, 67.5]	32.7 (40.6) 17.5 [4.8, 47.1]	42.2 (45.9) 29.7 [7.5, 63.1]	.0004*
**Recovery room stay** *(minutes)*	215 (199)	167 (122)	199 (178)	.0003*
**Average pain score**				
**In PACU**	3.5 [2, 5]	3.8 [2, 5.2]		0.512*
**On ward**	2.9 [1.8, 4.1]	2.7 [1.3, 3.8]		0.208*

^*^Wilcoxon, †chi-square, ‡Fisher exact (values reported as mean (STD) and median [IQR]

Time to meet discharge criteria from the post-anaesthesia care unit (PACU) was significantly reduced in both groups (215 ± 199 min vs. 167 ± 122 min, *p* = 0.003, + opioids intraoperative) and (253 ± 270 min vs. 167 ± 107 min, *p* = 0.0282, - opioids intraoperative). The duration of time until meeting discharge criteria from PACU was most prominent, saving an average of 51 min (221 ± 205 min vs. 170 ± 170 min, *p* < 0.0001; Table 5).

**Table 5:** Characteristics of patients receiving no intraoperative opioids (pre to post)

Matched** data**	**Pre (*n* = 68)**	**Post (*n* = 34)**	**Total (*n* = 102)**	***p*-value**
**Age**	62.0 (6.5)	61.9 (6.8)	62.0 (6.5)	0.9518*
**Race**				0.7487†
Black	24	12	36	
Other	7	2	9	
White	37	20	57	
**BMI**	28.7 (4.2)	28.5 (5.1)	28.7 (4.5)	0.8647*
**BSA**	2.1 (0.2)	2.1 (0.2)	2.13 (0.19)	0.8301‡
**ASA score**				0.8382†
1	1	0	1	
2	30	17	47	
3	36	17	53	
4	1	0	1	
**Smoker**	7	5	12	0.5280‡
**Chronic pain (current)**	11	5	16	1‡
**Chronic pain**	15	7	22	1‡
**Anxiety**	3	1	4	1‡
**Major depression**	2	0	2	0.5512‡
**Mood disorder**	0	0	0	N/A
**Drug use disorder**	0	1	1	0.3333‡
**Opioid tolerant**	0	0	0	N/A
**Sleep apnoea**	8	4	12	1‡
**Hospital LOS** *(days)*	1.7 (1.6)	1.3 (0.9)	1.6 (1.4)	.0583*
**Post-o**p** antiemetics**				
Any (Y/N)	35	9	44	.0163†
Average no. of doses	1.1 (1.7)	0.4 (0.9)	0.8 (1.5)	.0084*
**Post-o**p** opioids** *(OME)*	62.4 (62.9) 39.3 [14.85, 93.53]	32.7 (27.7) 36.45 [12.97, 54.08]	53.5 (55.1) 37.8 [14.4, 73.88]	0.1172*
**Recovery room stay** *(minutes)*	253 (270)	167 (105)	225 (233)	.0282*
**Average pain score**				
In PACU	3.8 [2, 5.2]	4 [2.9, 5.5]		0.425*
On ward	3.4 [2.1, 4.6]	3.4 [2.4, 4.5]		0.817*

^*^Wilcoxon, †chi-square, ‡Fisher exact (values reported as mean ± STD) and median [IQR]

Hospital LOS was significantly reduced (1.4 ± 1.3 days vs. 1.2 ± 0.8 days, *p* = 0.005). Both groups had reduced hospital LOS [(1.5 ± 1.3 days vs. 1.2 ± 0.8 days, *p* = 0.0047, + intraoperative opioids) and (1.7 ± 1.6 days vs. 1.3 ± 0.9 days, *p* = 0.0583, - intraoperative opioids)] (Tables 4 and 5). Although hospital LOS was statistically different between groups, it may not be clinically significant; however, the reduced number of bed hours may have contributed to the higher throughput of patients along the continuum and warrants further evaluation in prospective studies.

## Discussion

This study demonstrates that a multidisciplinary approach to reduce perioperative use of opioids can produce meaningful results when supported by all stakeholders along the perioperative continuum. Moreover, the ability to conduct major abdominal surgery without opioids results in significant reductions in both PACU and hospital LOS, reduced opioid use during hospital admission, and reduced incidence of post-operative nausea/vomiting most associated with opioid use, thereby improving recovery.

The growing use of opioids for the management of acute and chronic pain (Brennan et al. 2007), along with programmes promoting pain as ‘the fifth vital sign’, has been attributed to the increased use of opioid analgesics within the medical community over the past 10–15 years. The fifth vital sign campaign which employed a verbal, numerical pain scoring system (0 = no pain to 10 = intolerable pain) is now a mandatory part of the clinical assessment to establish the ‘adequacy of pain management’ used by most healthcare organizations in the USA, including the Joint Commission on Accreditation of Healthcare Organizations (JCAHO) (Ready et al. 1995). Routine measurement of the fifth vital sign has not, however, been shown to improve the quality of pain management (Mularski et al. 2006; Gan et al. 2014).

This controversial adoption of pain as the ‘fifth vital sign’ has led to significant increases in the average dosages of opioid analgesic medication administered in the early post-operative period after surgery (Aubrun et al. 2003) and increased the incidence of opioid-induced ‘over sedation’ cases by almost 150% (Vila et al. 2005; Lee et al. 2015). Of the patients experiencing life-threatening adverse reactions to opioid analgesics (e.g. respiratory and/or cardiac arrests), 94% had a documented decrease in their level of consciousness preceding the event (White 2017). In 2007, a review article (Brennan et al. 2007) by international experts in pain management further encouraged the more widespread use of opioid-containing analgesics by suggesting that ‘if only we [physicians and nurses] could overcome our “opiophobia”, we would improve pain management’.

The widespread use of opioids to relieve acute pain has unmasked the perverse effects of these analgesics in acute settings. In an editorial, Kehlet and White argued that ‘less may be more’ with respect to use of opioid (narcotic) analgesics (White and Kehlet 2007). These authors strongly argued for using non-opioid analgesics to reduce the dependence on oral and parenteral narcotic analgesics which would lessen the risk of opioid-related side effects. These well-known adverse effects include nausea-vomiting, dizziness, and pruritus, side effects which have been identified by patients as being most worrisome. So much so, that patients would accept experiencing more surgical pain rather than experience the opioid-related side effects of the following: nausea, vomiting, constipation, ileus, bladder dysfunction, pruritis, sedation, visual hallucinations, ventilatory depression, and long-term physical dependence and addiction liability (Gan et al. 2004).

Chronic opioid use is now one of the major social issues facing society today including misuse, abuse, addiction, and unintentional overdose resulting in death. Yet, in spite of all these and for unclear reasons, opioids remain the first line, most commonly used medications to treat pain (Lavand'homme and Steyaert 2017).

Even short-term use of potent opioid analgesics during the intraoperative period can actually aggravate pain due to opioid-induced hyperalgesia (i.e. acute tolerance) (Chia et al. 1999; Hayhurst and Durieux 2016; Zarate et al. 1999). It must be acknowledged that the prevalence of clinical opioid-induced hyperalgesia (OIH) during chronic opioid therapy remains unknown. A hyperalgesia state is often observed in former opioid abusers, especially those undergoing maintenance therapy with methadone, but these reports need to be interpreted cautiously, as opioid addicts’ personality may make determining if a hyperalgesia state exists difficult (Lavand'homme and Steyaert 2017).

Recent work published by Purdon et al. (Santa Cruz Mercado et al. 2023) suggests that reducing opioid use during surgery increases post-operative pain and increased opioid consumption. The authors fail to delineate if all analgesics or solely opioids were removed from the intraoperative period. We must stress here that opioid-free anaesthesia does NOT mean the omission of all classes of analgesics. Our care redesign prioritized non-opioid analgesics to be given first. Perhaps moving forward, the descriptor ‘opioid-free anaesthesia’ should be referred to as ‘non-opioid analgesic anaesthesia’ to highlight this important distinction.

Classic studies have demonstrated that the knowledgeable patient requires less analgesia in the post-operative period and at the same time experiences significantly less pain than the less-informed patient, and more recent investigations have supported the conclusion that preoperative information will aid coping, reduce preoperative anxiety, and may also enhance postsurgical recovery (Kehlet and Wilmore 2002).

Anaesthesiologists are leading experts in pain medicine, and through evidence-based implementations such as ERAS and multimodal anaesthesia, the specialty is helping to address the opioid crisis by reducing the amount of opioid used in the perioperative period whilst still maintaining adequate acute pain control. The time has come to change the foundations of our practice from that of an opioid-based one to that of a multimodal and multidisciplinary practice (Fig. 2), wherein analgesia is managed with non-opioid-based agents first, then layering on alternate non-opioid analgesics, and saving opioids as the capstone in analgesic management (Koepke et al. 2018).

Perioperative physicians and anaesthesiologists should continue to pursue evidence-based research to assist with the opioid epidemic from a broad, perioperative population health approach. We share the responsibility with the rest of the medical community not only to decrease the financial burden on society and on hospitals but also to assist in solving the epidemic, which has now become the number one accidental cause of death in the USA. In addition to the more pragmatic benefits of decreased PACU/hospital LOS, decreased opioid use and fewer opioid-related side effects improve patient outcomes, sometimes substantially — making an ORA/OFA approach favourable from that viewpoint.

### Limitations

We acknowledge several limitations of our study, one being its retrospective nature. Whilst our pathways essentially moved opioids in the position of a ‘rescue’ medication, the lower use of opioids during the post-operative period cannot solely be interpreted as less need for rescue medication, nor can its use suggest increased need. These questions are best answered via prospective study. Moreover, as pain scores were similar between cohorts, we do not know if the administration of opioids was given at the request of the patient or given based on beliefs and biases of members of the care team. Future studies will require not only capturing pain scores specifically but also patient’s expectations on pain, requests by patients for rescue medications, and data on provider’s interpretation and biases in pain management. Another critical limitation of our study is that we do not have access to long-term follow-up care and therefore are unable to report on the long-term use of opioids. Future work will include long-term follow-up to specifically address long-term opioid use as well as functional recovery in these patients.

## Conclusion

The collaborative efforts of all the members of the care teams that interact with surgical patients undergoing robotic prostatectomies, acting in a mindful, planned manner, were able to decrease opioid use, hospital, and PACU LOS and reduced opioid-related side effects. This teamwork allowed for consistency of practice, minimized variability, and allowed providers the ability to individually address needs as they arose within a guided framework of care. We believe that this type of approach is translatable across specialties. However, prospective studies are urgently needed to further evaluate the role of opioid-reduction pathways and opioid-free anaesthesia on a patient’s long-term outcomes.

### Supplementary Information

**Additional file 1: Table 1.** Opioid Free Pathway during the perioperative period. **Table 2.** Script elements to align expectations for OFA in Robotic Prostatectomies.

## Data Availability

Data sets and code used for the statistical analysis can be made available upon written request to the journal and authors.
