# Supplementary material for: A multidisciplinary opioid-reduction pathway for robotic prostatectomy: outcomes at year one
Source: Perioper Med (Lond). 2023 Aug 1;12:43. doi: 10.1186/s13741-023-00331-1 (PMC10391760; doi:10.1186/s13741-023-00331-1)
Supplement: Supplementary file 1 — Additional file 1: Table 1. Opioid Free Pathway during the perioperative period. Table 2. Script elements to align expectations for OFA in Robotic Prostatectomies. [file 13741_2023_331_MOESM1_ESM.docx]

**Supplemental Information**

|  |  | Duration of Surgery | | | | |
| --- | --- | --- | --- | --- | --- | --- |
| Drug |  | **Short (1-2 hours)** | **Long (> 2 hours)** | | **Notes** | |
| Pre-Operative *(day prior)* | | | | | | |
| Acetaminophen |  | 975 mg TID | | 975 mg TID | |  |
| Ibuprofen |  | 800 mg TID | | 800 mg TID | |  |
| Holding Room *(day of surgery)* | | | | | | |
| Celecoxib |  | 400 mg | 400 mg | |  | |
| Acetaminophen |  | 975 mg | 975 mg | |  | |
| Glycopyrrolate |  | 0.2 mg – 0.3 mg | 0.2 mg – 0.3 mg | |  | |
| Intraoperative | | | | | | |
| *Induction* |  |  |  | |  | |
| Ketamine |  | Bolus | Bolus ***and*** Infusion | | Bolus: 0.25 mg.kg^-1^ Infusion: 4 mcg.kg^-1^.min^-1^ | |
| Lidocaine |  | Bolus | Infusion | | Bolus: 1 mg.kg^-1^ Infusion: 1 mg.kg^-1^.hr^-1^ | |
| Dexmedetomidine |  |  | Bolus ***and*** Infusion | | Bolus: 1 mcg.kg^-1^ Infusion: 0.1 mcg.kg^-1^.hr^-1^ | |
| Magnesium |  | Bolus | Bolus ***and*** Infusion | | Bolus: 2 gm Infusion: 1 g.hr^-1^ | |
|  |  |  |  | |  | |
| *Analgesics (Administered based on Clinical Situation and Judgement of Provider at the time of closure)* | | | | | | |
| Ketorolac |  | Single Bolus | Single Bolus | | 15 - 30 mg  (If ≥ 6 hr since celecoxib administration) | |
| Hydromorphone |  | Bolus | Bolus | | 0.2 – 0.4 mg | |
| Morphine |  | Bolus | Bolus | | 0.06 – 0.1 mg.kg^-1^ | |
| PACU | |  | | | | |
| *Analgesics (Any 1 of the following; based on Clinical Situation and Judgement of Provider)* | | | | | | |
| Fentanyl |  | 25 mcg / dose | 25 mcg / dose | |  | |
| Hydromorphone |  | 0.2 – 0.4mg / dose | 0.2 – 0.4mg / dose | |  | |
| Morphine |  | 4 mg / dose | 4 mg / dose | |  | |
|  |  |  |  | |  | |
| *Antiemetics (Amy 1 of the following; based on Clinical Situation and Judgement of Provider)* | | | | | | |
| Ondansetron |  | 4 mg | 4 mg | |  | |
| Dexamethasone |  | 4 mg – 8 mg | 4 mg – 8 mg | |  | |
| Haloperidol |  | 1-2 mg | 1-2 mg | |  | |
| Diphenhydramine |  | 6.25 mg | 6.25 mg | |  | |

**Table 1:** Opioid Free Pathway during the perioperative period.

| Review / reinforce the origins of surgical pain and the expectations for pain control. |
| --- |
| Pain of 2-3 / 10 is expected and reasonable. |
| May have abdominal cramping sensations - “feels like doing 1,000 sit-ups”. |
| Opioids do not treat this type of pain very well and we will first use non-opioid medications to treat your pain more appropriately. |
| Pt may experience referred shoulder pain due to irritation of CO_2_ bubbles under the diaphragm. |
| Walking or changing positions will relieve this “aching pain”. |
| *Provide reassurance* usually resolves over 12-24 hours. |
| Opioid medications do not treat this discomfort well and may add to your side-effects. |
| In order to help prevent the side effects most commonly associated with opioid use: nausea/vomiting; difficulty urinating; constipation; confusion, we will treat your pain with non-opioid analgesics first, then if you need a little bit more, we can add opioid, use less and help avoid the unpleasant side effects. |

**Table 2:** Script elements to align expectations for OFA in Robotic Prostatectomies

Pathway for Opioid-Reduced/Opioid-Free Anaesthesia and Perioperative Care

*IF possible, begin Multi-modal Therapy the Day PRIOR to Surgery*

1. Tylenol 975 mg PO 3 times the day BEFORE surgery

**DAY of SURGERY**

Carbohydrate drink:

- Given to patient by preop screening to drink 1 hour before scheduled arrival time.
- If patient is coming from home, encourage drinking 20 ounces of Gatorade® ™ if CHO drink was not provided.

**PREOP HOLDING**

1. Holding Room nursing staff will
   1. IDENTIFY patient and initiate protocol
   2. DOCUMENT if CHO drink was taken and document time
2. Anaesthesiology Care Team to reinforce expectations and review education of the patient about expectations for pain and pain control.
   1. Expectation: Pain of 2-3/10 is expected and reasonable.
   2. Expectation: Pain of 5/10 would be the point to ask for additional medication
      (if patient were at home – they would self-treat at a 5/10)
   3. Expectation: May have ‘bloated’ sensation this is residual from insufflation – normal and resolves over 12 hours or less without treatment
   4. Education: Pt may experience referred shoulder pain due to irritation of CO_2_ bubble under diaphragm.
      1. Walking or changing positions will relieve this “aching pain”.
      2. Inform patient this will feel like an “over-use or strain” type of an ache.
      3. This resolves again over 12 hours or less.
3. Continue with Multimodal analgesia and antiemetics.
   These are to be given by Holding Room RN’s and confirmed by Attending anaesthesiologist.
   1. **Acetaminophen** 975 mg PO
   2. **Gabapentin** 600 mg PO
   3. **Celecoxib** 400 mg PO (HOLD IF PATIENT HAS RENAL IMPAIRMENT or CrCl < 30)
   4. **Aprepitant 40mg PO** for patients with proven history of post-operative nausea and/or vomiting
   5. **Glycopyrrolate** 0.2 mg – 0.3 mg IV to decrease secretions from Ketamine use.

**INTRAOPERATIVE**

1. **During Time-Out** –
   1. Discuss with surgeons placing the patient in 20° Trendelenburg position during trocar removal to completely vent CO_2_ gas from abdominal compartment. Encourage surgeons to compress abdomen during this manoeuvre to bring residual gas to umbilical trocar for venting.(Phelps, 2008)
   2. Discuss having surgeons infiltrate 0.25% Marcaine into the injection port sites during closing.
2. INDUCTION AGENTS: (Doses provided for reference)
   1. **Esmolol** 0.5 mg/kg IV Substituted for induction opioids
   2. **Lidocaine** 1.0-1.5 mg/kg IV – OMIT IF USING EXPAREL
   3. **Propofol** 1.0-1.5 mg/kg IV
   4. **Ketamine** 0.5 mg/kg IV Bolus. (i.e. – 80 kg patient would receive 40 mg)
   5. Paralytic of choice appropriate for situation/case
3. ANALGESIA DURING CASE:

(**DO NOT ADMINISTER OPIOIDS WITHOUT FIRST** Discussing with Attending)

- 1. Titrate inhalation agent to BIS of 40-60
  2. **Magnesium** 2g IV (Mavrommati et al., 2004; Albrecht et al., 2013; De Oliveira et al., 2013)
     (If patient has ESRD, is dialysis dependent, or Cr > 2.0 consider 1g)
  3. Run infusion of **Lidocaine** IV (1mg/kg/hr) – Consider continuing into PACU.
  4. Dexmedetomidine 12-20 mcg IV when closing

***Note: Refer to Anaesthesia Care Chart for recommendations for patients with Chronic Pain.***

1. HEMODYNAMIC MONITORING & GOALS – INTRAOPERATIVE:
   1. To reduce risk of increased intraocular pressure and postoperative blindness following steep Trendelenburg positioning, target higher MAP using arterial line (90-100mmHg) has been recommended.(Awad et al., 2009)
2. LOW FLOW ANAESTHESIA
   1. Keep flow at ≤ 1 L/min to help maintain body temp
   2. Recommend **Desflurane** in patients with BMI ≥ 30 (**Desflurane** has been shown to decrease the PACU length of stay when compared with Sevoflurane/Isoflurane)(Eger, 2002; 2008)
3. VENTILATION/OXYGENATION
   1. Maintain TV 6-8 ml/kg (based on Pt’s IDEAL body weight).
   2. Minimize FiO_2_ to 35%-40% to decrease absorptive atelectasis.
   3. Maintain PEEP at 7-8 cm H_2_O during insufflation.
   4. Increase PEEP as appropriate during steep Trendelenburg positioning
4. ANTIBIOTIC PROPHYLAXIS
   1. Ancef 2g-3g IV - Re-dosing as indicated.
   2. IF PENICILLIN ALLERGIC – Clindamycin 900mg IV+ ciprofloxacin 400mg IV.
      Re-dose clindamycin after 6 hours.
5. PONV prophylaxis
   1. Dexamethasone 4mg IV at start of case (may omit in diabetics)
   2. Ondansetron 4mg IV when closing
6. EMERGENCE from ANAESTHESIA: (Begin after undocking of Robot and closure of skin port sites).
   1. Ketorolac (15 mg – 30 mg IV based on hemodynamics & clinical assessment and IF ≥ 6 hrs since dosing of Celecoxib )
   2. **Hydromorphone** (up to 0.5 mg) IV PRN based on hemodynamics & clinical assessment.

**PACU**

1. PONV treatment
   1. Consider:

*(non-sedating options)*

- - 1. Haloperidol 1mg – 1.5 mg IV
    2. Metoclopramide 10 mg IV
    3. Ondansetron 4mg IV

*(may increase sedation)*

- - 1. Diphenhydramine 6.25 mg IV
    2. Promethazine 6.25 mg IV

1. Analgesia
   1. Discuss w/ Anaesthesia Attending prior to giving ONE OF THE FOLLOWING for breakthrough pain:
      1. Hydromorphone 0.2 mg – 0.4 mg
   2. Continue scheduled Gabapentin 100 mg on Q8 hour intervals
   3. Continue scheduled acetaminophen 975 mg at Q8 hour intervals

**Postoperative Floor Care as Per Surgical Team with Acute Pain Service Guidance**

1. Analgesia
2. Continue scheduled Gabapentin 100 mg on Q8 hour intervals
3. Continue scheduled acetaminophen 975 mg at Q8 hour intervals
4. Continue with ONE OF THE FOLLOWING
   1. Celebrex 100mg scheduled at Q12 hour intervals (Hold if CrCl < 30)
   2. Naproxen (Choose one of the following dosing schedules)
      1. 500 mg scheduled at Q12 hour intervals
      2. 250 mg scheduled at Q6-8-hour intervals
5. Oxycodone 5 mg – 10 mg Q4-6-hour intervals (per APS Recommendations (patient specific))

***References used for protocol development and consensus building:***

Albrecht E, Kirkham KR, Liu SS, Brull R. Peri-operative intravenous administration of magnesium sulphate and postoperative pain: a meta-analysis. Anaesthesia. 2013 Jan 1;68(1):79–90.

Awad H, Santilli S, Ohr M, Roth A, Yan W, Fernandez S, et al. The effects of steep trendelenburg positioning on intraocular pressure during robotic radical prostatectomy. Anesthesia & Analgesia. 2009 Aug 1;109(2):473–8.

Carollo DS, Nossaman BD. Dexmedetomidine: a review of clinical applications. Current opinion in …. 2008.

De Oliveira GS, Castro-Alves LJ, Khan JH, McCarthy RJ. Perioperative systemic magnesium to minimize postoperative pain: a meta-analysis of randomized controlled trials. Anesthesiology. 2013 Jul;119(1):178–90.

Eger EI. Clinical Implications of Inhaled Anesthetic Pharmacology. ASA Refresher Courses in Anesthesiology. 2002.

Eger EI. Pharmacology of Isoflurane Compared to Other General Anesthetics. ASA Refresher Courses in Anesthesiology. 2008 Oct 13;9:1–24.

Grewal A. Dexmedetomidine: New avenues. Journal of anaesthesiology, clinical pharmacology. 2011 Jul 1;27(3):297–302. PMCID: PMC3161450

Mavrommati PD, Gabopoulou ZT, Papadimos CN, Petsikopoulos MG, Vrettou VA, Konstantinidou MG, et al. The perioperative infusion of low doses of magnesium sulfate reduces analgesic requirements in patients undergoing abdominal hernioplasty. Acute Pain. 2004 Apr;5(3-4):81–7.

Pestieau SR, Quezado ZMN, Johnson YJ, Anderson JL, Cheng YI, McCarter RJ, et al. High-dose dexmedetomidine increases the opioid-free interval and decreases opioid requirement after tonsillectomy in children. Can J Anaesth. Springer-Verlag; 2011;58(6):540–50.

Phelps P, Cakmakkaya OS, Apfel CC, Radke OC. A simple clinical maneuver to reduce laparoscopy-induced shoulder pain: a randomized controlled trial. Obstetrics & Gynecology [Internet]. 2008 May;111(5):1155–60. Retrieved from: http://content.wkhealth.com/linkback/openurl?sid=WKPTLP:landingpage&an=00006250-200805000-00021
